# Texas State Medical Association—Arrangements for the Coming Meeting, April 22

**Published:** 1890-03

**Authors:** 


### TEXAS STATE MEDICAL ASSOCIATION.

### Arrangements for the Coming Meeting, April 22.

The JOURNAL has been favored, by the courteous Chairman of the Arrangement Committee, with the following memoranda of what has been done towards receiving the delegates to the Twenty-second Annual Convention of the Texas State Medical Association:

A meeting, in response to a call from Chairman Burts, was held in his office, and the following committees were appointed, Dr. Grammer acting as secretary, Dr. Burts presiding. It will be seen that the Committee of Arrangements decided to entertain the Association at a ball and banquet, notwithstanding the protest of certain members.

On Banquet—Drs. H. S. Broiles, W. A. Adams, J. M. Mullins, J. C. McCoy and J. N. McKnight.

On Ball—Drs. W. A. Duringer, J. W. Irion, L. B. Slaughter, C. A. Parker and W. Beverly West.

On Transportation—Drs. W. A. Adams, J. T. Field and W. P. Burts.

On Finance—Drs. E. J. Beall, M. Matkin, F. D. Thompson, Frank Gray, Wm. R. Howard, I. L. VanZandt and Frank Mullins.

On Programme—Drs. A. P. Brown, R. B. Grammer, W. A. Adams, J. Anderson and J. B. McLean.

On Music and Hall—Drs. J. L. Cooper, R. J. Wilson, A. E. Graham, W. Beverly West, I. M. Darter, G. W. Magruder and J. R. Adams.

On Printing and Badges—Drs. L. B. Slaughter, W. A. Duringer, R. B. Grammer and C. Guy Reily.

Dr. W. P. Burts was unanimously chosen to deliver the address of welcome on behalf of the local medical profession.

It was resolved to request the Mayor of Fort Worth to deliver the address of welcome on behalf of the citizens.

On adjournment it was agreed to meet again at the same place to further the work.

